# *N*,*N*-Diethyl-4-[1-phenyl-3-(pyridin-2-yl)-4,5-di­hydro-1*H*-pyrazol-5-yl]aniline

**DOI:** 10.1107/S1600536813019879

**Published:** 2013-07-27

**Authors:** Ying-Zhong Zhu, Hui Wang, Ping-Ping Sun, Yu-Peng Tian

**Affiliations:** a Department of Chemistry, Anhui University, Hefei 230039, People’s Republic of China; b Key Laboratory of Functional Inorganic Materials Chemistry, Hefei 230039, People’s Republic of China

## Abstract

In the title mol­ecule, C_24_H_26_N_4_, the pyrazoline ring assumes an envelope conformation with the aniline-bearing C atom at the flap position. The benzene ring and the pyridine ring form with the pyrazoline ring dihedral angles of 4.53 (1) and 6.26 (1)°, respectively. In turn, the aniline group is nearly perpendicular to the pyrazoline ring [dihedral angle = 79.96 (1)°]. The ethyl groups of the di­ethyl­amine substituent are disordered over two sets of sites, with an occupancy ratio of 0.624 (8):0.376 (8).

## Related literature

For background to the design and synthesis of the title compound and for related structures, see: Chen *et al.* (2008 ▶); Dong *et al.* (2010 ▶); Guo *et al.* (2010 ▶); Liu *et al.* (2010 ▶).
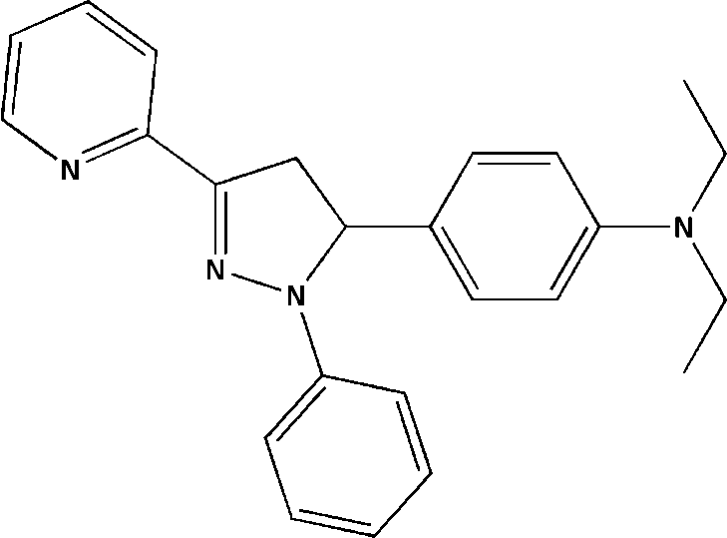


## Experimental

### 

#### Crystal data

C_24_H_26_N_4_
*M* *_r_* = 370.49
Monoclinic, 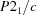

*a* = 14.902 (5) Å
*b* = 11.314 (5) Å
*c* = 12.479 (5) Å
β = 94.644 (5)°
*V* = 2097.1 (14) Å^3^
*Z* = 4
Mo *K*α radiation
μ = 0.07 mm^−1^
*T* = 296 K
0.30 × 0.20 × 0.20 mm

#### Data collection

Bruker SMART APEX CCD diffractometer
Absorption correction: multi-scan (*SADABS*; Bruker, 2000 ▶) *T*_min_ = 0.979, *T*_max_ = 0.986
14365 measured reflections
3678 independent reflections
2511 reflections with *I* > 2σ(*I*)
*R*_int_ = 0.038

#### Refinement

*R*[*F* ^2^ > 2σ(*F* ^2^)] = 0.073
*wR*(*F* ^2^) = 0.241
*S* = 1.06
3678 reflections
294 parameters
507 restraints
H-atom parameters constrained
Δρ_max_ = 0.37 e Å^−3^
Δρ_min_ = −0.52 e Å^−3^

### 

Data collection: *SMART* (Bruker, 2002 ▶); cell refinement: *SAINT* (Bruker, 2002 ▶); data reduction: *SAINT*; program(s) used to solve structure: *SHELXS97* (Sheldrick, 2008 ▶); program(s) used to refine structure: *SHELXL97* (Sheldrick, 2008 ▶); molecular graphics: *SHELXTL* (Sheldrick, 2008 ▶); software used to prepare material for publication: *SHELXTL*.

## Supplementary Material

Crystal structure: contains datablock(s) I, global. DOI: 10.1107/S1600536813019879/gk2584sup1.cif

Structure factors: contains datablock(s) I. DOI: 10.1107/S1600536813019879/gk2584Isup2.hkl

Supplementary material file. DOI: 10.1107/S1600536813019879/gk2584Isup3.cml

Additional supplementary materials:  crystallographic information; 3D view; checkCIF report

## Figures and Tables

**Fig. 1.**
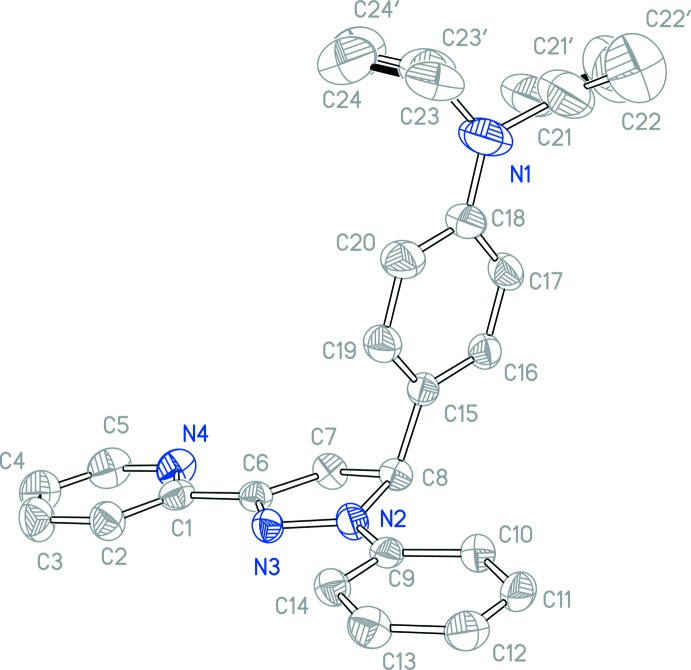
The molecular structure of the title compound with the atom-numbering scheme. Displacement ellipsoids are drawn at the 30% probability level. H atoms have been omitted.

## supplementary crystallographic information

### Comment

The derivatives of the title compound are often used in medicinal chemistry and biochemistry (Guo *et al.* 2010; Liu *et al.* 2010). Herewith , in this study, we report the crystal structure of the title compound.

The pyrazoline ring is nearly coplanar to the benzene ring and the pyridine ring, with the dihedral angles of 4.53 (1)° and 6.26 (1)°, respectively (Fig.1). The anilinegroup is almost perpendicular to the pyrazoline ring, with a dihedral angle of 79.96 (1)°. The ethyl groups of the N,N-diethylaniline unit are disordered.

### Experimental

A mixture of 3-(4-(diethylamino)phenyl)-1-(pyridin-2-yl)prop-2-en-1-one (2.8 g, 10 mmol), phenylhydrazine (1.5 ml) and acetic acid (10 ml) was refluxed for 4 h, cooled and Na_2_CO_3_ was added to modify the pH under vigorously stirring. to pH = 7. The mixture was washed with dichloromethane and the solution was evaporated and the crude product was obtained and purified by flash column chromatography (silica, 10:1 petroleum ether: ethyl acetate) to obtain light yellow crystals. Yiels: 40%. ^1^H NMR (400 MHz, DMSO-d^6^) 1.03 (t, 6H), 3.11 (q, 1H), 3.25 (q, 4H), 3.86 (q, 1H) 5.37 (q, 1H), 6.57 (d, 2H), 6.74 (t, 1H), 7.06(q, 4H), 7,17(t, 2H), 7.30 (t, 1H), 7.82 (t, 1H) 8.09 (d, 1H), 8.54 (d, 1H).

### Refinement

All hydrogen atoms were placed in geometrically idealized positions and constrained to ride on their parent atoms, with C—H = 0.93 Å and *U*_iso_(H) = 1.2 *U*_eq_. The ethyl groups of the N,N-diethylamino group are disordered over two positions. Restraints were imposed on C-C and C-N distances and displacement parameters in these groups.

### Crystal data

**Table:**

C_24_H_26_N_4_	*F*(000) = 792
*M**_r_* = 370.49	*D*_x_ = 1.173 Mg m^−^^3^
Monoclinic, *P*2_1_/*c*	Melting point: 391 K
Hall symbol: -P 2ybc	Mo *K*α radiation, λ = 0.71069 Å
*a* = 14.902 (5) Å	Cell parameters from 3174 reflections
*b* = 11.314 (5) Å	θ = 2.4–23.1°
*c* = 12.479 (5) Å	µ = 0.07 mm^−^^1^
β = 94.644 (5)°	*T* = 296 K
*V* = 2097.1 (14) Å^3^	Block, yellow
*Z* = 4	0.30 × 0.20 × 0.20 mm

### Data collection

**Table:**

Bruker SMART APEX CCD diffractometer	3678 independent reflections
Radiation source: fine-focus sealed tube	2511 reflections with *I* > 2σ(*I*)
Graphite monochromator	*R*_int_ = 0.038
φ and ω scans	θ_max_ = 25.0°, θ_min_ = 2.3°
Absorption correction: multi-scan (*SADABS*; Bruker, 2000)	*h* = −17→17
*T*_min_ = 0.979, *T*_max_ = 0.986	*k* = −13→13
14365 measured reflections	*l* = −14→14

### Refinement

**Table:**

Refinement on *F*^2^	Primary atom site location: structure-invariant direct methods
Least-squares matrix: full	Secondary atom site location: difference Fourier map
*R*[*F*^2^ > 2σ(*F*^2^)] = 0.073	Hydrogen site location: inferred from neighbouring sites
*wR*(*F*^2^) = 0.241	H-atom parameters constrained
*S* = 1.06	*w* = 1/[σ^2^(*F*_o_^2^) + (0.1447*P*)^2^ + 0.5666*P*] where *P* = (*F*_o_^2^ + 2*F*_c_^2^)/3
3678 reflections	(Δ/σ)_max_ = 0.002
294 parameters	Δρ_max_ = 0.37 e Å^−^^3^
507 restraints	Δρ_min_ = −0.52 e Å^−^^3^

### Special details

**Table:**

Geometry. All e.s.d.'s (except the e.s.d. in the dihedral angle between two l.s. planes) are estimated using the full covariance matrix. The cell e.s.d.'s are taken into account individually in the estimation of e.s.d.'s in distances, angles and torsion angles; correlations between e.s.d.'s in cell parameters are only used when they are defined by crystal symmetry. An approximate (isotropic) treatment of cell e.s.d.'s is used for estimating e.s.d.'s involving l.s. planes.
Refinement. Refinement of *F*^2^ against ALL reflections. The weighted *R*-factor *wR* and goodness of fit *S* are based on *F*^2^, conventional *R*-factors *R* are based on *F*, with *F* set to zero for negative *F*^2^. The threshold expression of *F*^2^ > σ(*F*^2^) is used only for calculating *R*-factors(gt) *etc*. and is not relevant to the choice of reflections for refinement. *R*-factors based on *F*^2^ are statistically about twice as large as those based on *F*, and *R*- factors based on ALL data will be even larger.

### Fractional atomic coordinates and isotropic or equivalent isotropic displacement parameters (Å^2^)

**Table:**

	*x*	*y*	*z*	*U*_iso_*/*U*_eq_	Occ. (<1)
C1	0.3073 (2)	−0.2820 (3)	0.4677 (3)	0.0539 (7)	
C2	0.3140 (2)	−0.3449 (3)	0.5624 (3)	0.0662 (9)	
H2	0.3316	−0.3074	0.6271	0.079*	
C3	0.2946 (3)	−0.4632 (3)	0.5606 (4)	0.0815 (10)	
H3	0.3000	−0.5071	0.6239	0.098*	
C4	0.2674 (3)	−0.5163 (4)	0.4651 (4)	0.0864 (11)	
H4	0.2538	−0.5965	0.4616	0.104*	
C5	0.2611 (3)	−0.4473 (4)	0.3752 (4)	0.0821 (11)	
H5	0.2423	−0.4835	0.3103	0.099*	
C6	0.32889 (19)	−0.1562 (3)	0.4637 (2)	0.0504 (7)	
C7	0.3245 (2)	−0.0819 (3)	0.3641 (2)	0.0561 (8)	
H7A	0.2659	−0.0876	0.3245	0.067*	
H7B	0.3706	−0.1047	0.3175	0.067*	
C8	0.3415 (2)	0.0433 (3)	0.4096 (2)	0.0519 (7)	
H8	0.3888	0.0822	0.3723	0.062*	
C9	0.40911 (18)	0.0948 (2)	0.5983 (2)	0.0485 (7)	
C10	0.4309 (2)	0.2102 (3)	0.5699 (3)	0.0597 (8)	
H10	0.4198	0.2357	0.4992	0.072*	
C11	0.4691 (2)	0.2863 (3)	0.6473 (3)	0.0625 (8)	
H11	0.4848	0.3623	0.6275	0.075*	
C12	0.4846 (2)	0.2523 (3)	0.7527 (3)	0.0633 (8)	
H12	0.5106	0.3043	0.8039	0.076*	
C13	0.4608 (2)	0.1400 (3)	0.7814 (3)	0.0610 (8)	
H13	0.4703	0.1164	0.8528	0.073*	
C14	0.4230 (2)	0.0613 (3)	0.7055 (2)	0.0529 (7)	
H14	0.4069	−0.0142	0.7264	0.064*	
C15	0.2580 (2)	0.1196 (3)	0.4035 (2)	0.0504 (7)	
C16	0.2344 (2)	0.1879 (3)	0.3139 (3)	0.0572 (8)	
H16	0.2736	0.1927	0.2597	0.069*	
C17	0.1544 (2)	0.2488 (3)	0.3028 (3)	0.0670 (9)	
H17	0.1410	0.2942	0.2415	0.080*	
C18	0.0926 (3)	0.2446 (3)	0.3813 (3)	0.0760 (9)	
C19	0.1985 (2)	0.1174 (3)	0.4831 (3)	0.0656 (9)	
H19	0.2132	0.0738	0.5452	0.079*	
C20	0.1182 (3)	0.1780 (4)	0.4727 (3)	0.0767 (10)	
H20	0.0801	0.1744	0.5280	0.092*	
C21	−0.0287 (6)	0.3410 (9)	0.2615 (7)	0.1169 (19)	0.624 (8)
H21A	0.0049	0.3045	0.2069	0.140*	0.624 (8)
H21B	−0.0903	0.3128	0.2514	0.140*	0.624 (8)
C22	−0.0282 (8)	0.4663 (10)	0.2471 (10)	0.151 (3)	0.624 (8)
H22A	−0.0691	0.5022	0.2931	0.227*	0.624 (8)
H22B	−0.0465	0.4849	0.1735	0.227*	0.624 (8)
H22C	0.0315	0.4961	0.2651	0.227*	0.624 (8)
C23	−0.0521 (6)	0.2931 (9)	0.4622 (8)	0.1072 (18)	0.624 (8)
H23A	−0.0175	0.3015	0.5310	0.129*	0.624 (8)
H23B	−0.0961	0.3564	0.4556	0.129*	0.624 (8)
C24	−0.1004 (7)	0.1750 (9)	0.4583 (10)	0.127 (2)	0.624 (8)
H24A	−0.1462	0.1748	0.3995	0.190*	0.624 (8)
H24B	−0.1277	0.1627	0.5245	0.190*	0.624 (8)
H24C	−0.0580	0.1129	0.4483	0.190*	0.624 (8)
C21'	−0.0141 (8)	0.4066 (13)	0.2996 (14)	0.118 (2)	0.376 (8)
H21C	0.0205	0.3986	0.2373	0.142*	0.376 (8)
H21D	−0.0766	0.3947	0.2739	0.142*	0.376 (8)
C22'	−0.0068 (15)	0.5244 (13)	0.3306 (17)	0.146 (3)	0.376 (8)
H22D	−0.0077	0.5736	0.2679	0.219*	0.376 (8)
H22E	0.0488	0.5360	0.3739	0.219*	0.376 (8)
H22F	−0.0563	0.5449	0.3715	0.219*	0.376 (8)
C23'	−0.0689 (11)	0.2690 (15)	0.4247 (14)	0.108 (2)	0.376 (8)
H23C	−0.1125	0.3326	0.4158	0.129*	0.376 (8)
H23D	−0.0518	0.2612	0.5010	0.129*	0.376 (8)
C24'	−0.1138 (11)	0.1567 (14)	0.3859 (17)	0.128 (3)	0.376 (8)
H24D	−0.1529	0.1725	0.3226	0.191*	0.376 (8)
H24E	−0.1483	0.1253	0.4410	0.191*	0.376 (8)
H24F	−0.0689	0.1002	0.3692	0.191*	0.376 (8)
N1	0.0113 (3)	0.3032 (4)	0.3708 (4)	0.1077 (11)	
N2	0.37522 (17)	0.0154 (2)	0.5210 (2)	0.0555 (7)	
N3	0.35871 (16)	−0.0994 (2)	0.5487 (2)	0.0502 (7)	
N4	0.2797 (2)	−0.3311 (3)	0.3729 (2)	0.0696 (8)	

### Atomic displacement parameters (Å^2^)

**Table:**

	*U*^11^	*U*^22^	*U*^33^	*U*^12^	*U*^13^	*U*^23^
C1	0.0467 (15)	0.0507 (15)	0.0640 (17)	0.0005 (12)	0.0025 (13)	−0.0102 (13)
C2	0.0719 (19)	0.0515 (16)	0.0733 (18)	−0.0062 (14)	−0.0061 (15)	−0.0017 (14)
C3	0.093 (2)	0.0560 (18)	0.093 (2)	−0.0094 (17)	−0.0055 (19)	0.0011 (16)
C4	0.091 (2)	0.060 (2)	0.107 (2)	−0.0093 (18)	0.000 (2)	−0.0174 (17)
C5	0.085 (2)	0.070 (2)	0.091 (2)	−0.0079 (19)	0.0039 (19)	−0.0321 (18)
C6	0.0451 (14)	0.0499 (15)	0.0558 (16)	0.0017 (12)	0.0025 (12)	−0.0043 (12)
C7	0.0558 (16)	0.0582 (16)	0.0542 (16)	0.0055 (13)	0.0029 (13)	−0.0026 (13)
C8	0.0491 (15)	0.0538 (16)	0.0528 (16)	−0.0022 (12)	0.0035 (12)	0.0057 (13)
C9	0.0431 (14)	0.0415 (14)	0.0601 (15)	0.0028 (11)	−0.0010 (12)	−0.0026 (12)
C10	0.0593 (17)	0.0488 (16)	0.0701 (17)	−0.0025 (13)	0.0002 (14)	0.0051 (13)
C11	0.0580 (17)	0.0444 (15)	0.0845 (19)	−0.0060 (13)	0.0023 (15)	−0.0043 (14)
C12	0.0580 (17)	0.0526 (16)	0.0785 (18)	0.0024 (13)	0.0012 (15)	−0.0182 (15)
C13	0.0638 (17)	0.0558 (16)	0.0628 (17)	0.0054 (14)	0.0012 (14)	−0.0082 (14)
C14	0.0549 (15)	0.0440 (15)	0.0595 (16)	0.0041 (12)	0.0027 (13)	−0.0022 (12)
C15	0.0514 (14)	0.0491 (15)	0.0505 (14)	−0.0032 (12)	0.0020 (11)	0.0032 (12)
C16	0.0616 (16)	0.0541 (16)	0.0554 (16)	−0.0025 (13)	0.0011 (13)	0.0052 (13)
C17	0.0693 (18)	0.0611 (18)	0.0687 (18)	0.0042 (15)	−0.0066 (14)	0.0106 (15)
C18	0.0663 (17)	0.0719 (19)	0.089 (2)	0.0109 (15)	0.0018 (15)	0.0032 (17)
C19	0.0621 (17)	0.0722 (19)	0.0636 (17)	0.0075 (15)	0.0114 (14)	0.0130 (15)
C20	0.0684 (19)	0.085 (2)	0.0787 (19)	0.0111 (16)	0.0192 (16)	0.0080 (17)
C21	0.106 (3)	0.121 (3)	0.123 (3)	0.024 (3)	0.004 (2)	0.006 (3)
C22	0.164 (4)	0.140 (4)	0.150 (4)	0.008 (3)	0.005 (3)	0.021 (3)
C23	0.090 (3)	0.113 (3)	0.119 (3)	0.024 (2)	0.009 (2)	−0.002 (3)
C24	0.114 (4)	0.133 (4)	0.134 (4)	0.001 (3)	0.012 (3)	0.001 (3)
C21'	0.112 (3)	0.119 (3)	0.123 (3)	0.022 (3)	0.003 (3)	0.013 (3)
C22'	0.153 (5)	0.135 (4)	0.149 (5)	0.003 (4)	0.008 (4)	0.005 (3)
C23'	0.094 (3)	0.114 (3)	0.116 (3)	0.019 (3)	0.013 (3)	−0.001 (3)
C24'	0.123 (4)	0.130 (4)	0.130 (5)	0.000 (3)	0.013 (4)	−0.002 (4)
N1	0.0858 (18)	0.116 (2)	0.121 (2)	0.0296 (16)	0.0086 (16)	0.0113 (17)
N2	0.0629 (16)	0.0440 (14)	0.0571 (15)	−0.0043 (11)	−0.0099 (12)	0.0060 (11)
N3	0.0510 (14)	0.0407 (14)	0.0578 (15)	−0.0016 (11)	−0.0027 (11)	0.0016 (11)
N4	0.0717 (19)	0.0666 (19)	0.0706 (19)	−0.0078 (14)	0.0057 (14)	−0.0201 (15)

### Geometric parameters (Å, º)

**Table:**

C1—N4	1.341 (4)	C17—H17	0.9300
C1—C2	1.377 (5)	C18—N1	1.378 (5)
C1—C6	1.461 (4)	C18—C20	1.394 (5)
C2—C3	1.370 (5)	C19—C20	1.377 (5)
C2—H2	0.9300	C19—H19	0.9300
C3—C4	1.366 (6)	C20—H20	0.9300
C3—H3	0.9300	C21—C22	1.429 (12)
C4—C5	1.365 (6)	C21—N1	1.506 (9)
C4—H4	0.9300	C21—H21A	0.9700
C5—N4	1.345 (5)	C21—H21B	0.9700
C5—H5	0.9300	C22—H22A	0.9600
C6—N3	1.288 (4)	C22—H22B	0.9600
C6—C7	1.498 (4)	C22—H22C	0.9600
C7—C8	1.540 (4)	C23—C24	1.516 (12)
C7—H7A	0.9700	C23—N1	1.543 (10)
C7—H7B	0.9700	C23—H23A	0.9700
C8—N2	1.473 (4)	C23—H23B	0.9700
C8—C15	1.511 (4)	C24—H24A	0.9600
C8—H8	0.9800	C24—H24B	0.9600
C9—N2	1.383 (4)	C24—H24C	0.9600
C9—C14	1.390 (4)	C21'—C22'	1.390 (16)
C9—C10	1.398 (4)	C21'—N1	1.499 (12)
C10—C11	1.382 (5)	C21'—H21C	0.9700
C10—H10	0.9300	C21'—H21D	0.9700
C11—C12	1.372 (5)	C22'—H22D	0.9600
C11—H11	0.9300	C22'—H22E	0.9600
C12—C13	1.374 (5)	C22'—H22F	0.9600
C12—H12	0.9300	C23'—N1	1.470 (14)
C13—C14	1.386 (4)	C23'—C24'	1.498 (17)
C13—H13	0.9300	C23'—H23C	0.9700
C14—H14	0.9300	C23'—H23D	0.9700
C15—C16	1.381 (4)	C24'—H24D	0.9600
C15—C19	1.383 (4)	C24'—H24E	0.9600
C16—C17	1.375 (5)	C24'—H24F	0.9600
C16—H16	0.9300	N2—N3	1.371 (3)
C17—C18	1.398 (5)		
			
N4—C1—C2	122.6 (3)	N1—C18—C17	122.8 (4)
N4—C1—C6	115.2 (3)	C20—C18—C17	116.1 (3)
C2—C1—C6	122.1 (3)	C20—C19—C15	121.7 (3)
C3—C2—C1	119.4 (3)	C20—C19—H19	119.1
C3—C2—H2	120.3	C15—C19—H19	119.1
C1—C2—H2	120.3	C19—C20—C18	121.7 (3)
C4—C3—C2	119.4 (4)	C19—C20—H20	119.2
C4—C3—H3	120.3	C18—C20—H20	119.2
C2—C3—H3	120.3	C22—C21—N1	112.9 (8)
C5—C4—C3	117.5 (4)	C22—C21—H21A	109.0
C5—C4—H4	121.2	N1—C21—H21A	109.0
C3—C4—H4	121.2	C22—C21—H21B	109.0
N4—C5—C4	125.2 (4)	N1—C21—H21B	109.0
N4—C5—H5	117.4	H21A—C21—H21B	107.8
C4—C5—H5	117.4	C24—C23—N1	111.0 (7)
N3—C6—C1	121.4 (3)	C24—C23—H23A	109.4
N3—C6—C7	113.1 (3)	N1—C23—H23A	109.4
C1—C6—C7	125.4 (3)	C24—C23—H23B	109.4
C6—C7—C8	102.4 (2)	N1—C23—H23B	109.4
C6—C7—H7A	111.3	H23A—C23—H23B	108.0
C8—C7—H7A	111.3	C22'—C21'—N1	124.9 (14)
C6—C7—H7B	111.3	C22'—C21'—H21C	106.1
C8—C7—H7B	111.3	N1—C21'—H21C	106.1
H7A—C7—H7B	109.2	C22'—C21'—H21D	106.1
N2—C8—C15	112.8 (2)	N1—C21'—H21D	106.1
N2—C8—C7	100.6 (2)	H21C—C21'—H21D	106.3
C15—C8—C7	113.4 (2)	C21'—C22'—H22D	109.5
N2—C8—H8	109.9	C21'—C22'—H22E	109.5
C15—C8—H8	109.9	H22D—C22'—H22E	109.5
C7—C8—H8	109.9	C21'—C22'—H22F	109.5
N2—C9—C14	120.8 (3)	H22D—C22'—H22F	109.5
N2—C9—C10	120.6 (3)	H22E—C22'—H22F	109.5
C14—C9—C10	118.6 (3)	N1—C23'—C24'	115.7 (12)
C11—C10—C9	119.7 (3)	N1—C23'—H23C	108.4
C11—C10—H10	120.1	C24'—C23'—H23C	108.4
C9—C10—H10	120.1	N1—C23'—H23D	108.4
C12—C11—C10	121.5 (3)	C24'—C23'—H23D	108.4
C12—C11—H11	119.2	H23C—C23'—H23D	107.4
C10—C11—H11	119.2	C23'—C24'—H24D	109.5
C11—C12—C13	118.8 (3)	C23'—C24'—H24E	109.5
C11—C12—H12	120.6	H24D—C24'—H24E	109.5
C13—C12—H12	120.6	C23'—C24'—H24F	109.5
C12—C13—C14	121.0 (3)	H24D—C24'—H24F	109.5
C12—C13—H13	119.5	H24E—C24'—H24F	109.5
C14—C13—H13	119.5	C18—N1—C23'	124.9 (8)
C13—C14—C9	120.2 (3)	C18—N1—C21'	127.5 (6)
C13—C14—H14	119.9	C23'—N1—C21'	107.5 (9)
C9—C14—H14	119.9	C18—N1—C21	120.2 (5)
C16—C15—C19	117.1 (3)	C23'—N1—C21	102.2 (8)
C16—C15—C8	121.0 (3)	C18—N1—C23	118.7 (5)
C19—C15—C8	121.7 (3)	C21'—N1—C23	110.8 (7)
C17—C16—C15	121.6 (3)	C21—N1—C23	118.0 (6)
C17—C16—H16	119.2	N3—N2—C9	120.2 (2)
C15—C16—H16	119.2	N3—N2—C8	112.7 (2)
C16—C17—C18	121.8 (3)	C9—N2—C8	126.6 (2)
C16—C17—H17	119.1	C6—N3—N2	108.8 (2)
C18—C17—H17	119.1	C1—N4—C5	115.8 (3)
N1—C18—C20	121.2 (4)		
			
N4—C1—C2—C3	2.0 (5)	C20—C18—N1—C21'	158.4 (10)
C6—C1—C2—C3	−178.6 (3)	C17—C18—N1—C21'	−21.0 (11)
C1—C2—C3—C4	−1.1 (6)	C20—C18—N1—C21	−160.3 (6)
C2—C3—C4—C5	0.1 (6)	C17—C18—N1—C21	20.3 (8)
C3—C4—C5—N4	0.2 (7)	C20—C18—N1—C23	−0.5 (7)
N4—C1—C6—N3	−177.9 (3)	C17—C18—N1—C23	−179.9 (5)
C2—C1—C6—N3	2.7 (5)	C24'—C23'—N1—C18	−69.5 (17)
N4—C1—C6—C7	−1.5 (4)	C24'—C23'—N1—C21'	107.3 (16)
C2—C1—C6—C7	179.1 (3)	C24'—C23'—N1—C21	71.6 (16)
N3—C6—C7—C8	−10.6 (3)	C24'—C23'—N1—C23	−150 (4)
C1—C6—C7—C8	172.7 (3)	C22'—C21'—N1—C18	−93.3 (15)
C6—C7—C8—N2	14.0 (3)	C22'—C21'—N1—C23'	90.1 (16)
C6—C7—C8—C15	−106.7 (3)	C22'—C21'—N1—C21	176 (2)
N2—C9—C10—C11	−176.6 (3)	C22'—C21'—N1—C23	67.0 (16)
C14—C9—C10—C11	2.8 (5)	C22—C21—N1—C18	−110.5 (8)
C9—C10—C11—C12	−1.5 (5)	C22—C21—N1—C23'	106.1 (10)
C10—C11—C12—C13	−0.3 (5)	C22—C21—N1—C21'	2.9 (10)
C11—C12—C13—C14	0.8 (5)	C22—C21—N1—C23	89.6 (9)
C12—C13—C14—C9	0.5 (5)	C24—C23—N1—C18	−77.2 (8)
N2—C9—C14—C13	177.0 (3)	C24—C23—N1—C23'	35 (2)
C10—C9—C14—C13	−2.4 (4)	C24—C23—N1—C21'	120.6 (10)
N2—C8—C15—C16	157.4 (3)	C24—C23—N1—C21	83.1 (9)
C7—C8—C15—C16	−89.1 (3)	C14—C9—N2—N3	−3.0 (4)
N2—C8—C15—C19	−27.6 (4)	C10—C9—N2—N3	176.4 (3)
C7—C8—C15—C19	85.9 (4)	C14—C9—N2—C8	167.8 (3)
C19—C15—C16—C17	−1.5 (5)	C10—C9—N2—C8	−12.8 (5)
C8—C15—C16—C17	173.7 (3)	C15—C8—N2—N3	106.4 (3)
C15—C16—C17—C18	−0.3 (5)	C7—C8—N2—N3	−14.7 (3)
C16—C17—C18—N1	−178.7 (4)	C15—C8—N2—C9	−65.0 (4)
C16—C17—C18—C20	1.9 (6)	C7—C8—N2—C9	173.9 (3)
C16—C15—C19—C20	1.6 (5)	C1—C6—N3—N2	178.5 (3)
C8—C15—C19—C20	−173.6 (3)	C7—C6—N3—N2	1.7 (3)
C15—C19—C20—C18	0.1 (6)	C9—N2—N3—C6	−179.1 (3)
N1—C18—C20—C19	178.8 (4)	C8—N2—N3—C6	8.9 (3)
C17—C18—C20—C19	−1.8 (6)	C2—C1—N4—C5	−1.7 (5)
C20—C18—N1—C23'	−25.5 (10)	C6—C1—N4—C5	178.9 (3)
C17—C18—N1—C23'	155.1 (9)	C4—C5—N4—C1	0.6 (6)
